# Expression of tissue inhibitor of matrix metalloproteinases (TIMP)-3 protein in invasive breast carcinoma: Relation to tumor phenotype and clinical outcome

**DOI:** 10.1186/bcr1607

**Published:** 2006-10-10

**Authors:** Eleni Mylona, Christina Magkou, Ioanna Giannopoulou, George Agrogiannis, Sofia Markaki, Antonios Keramopoulos, Lydia Nakopoulou

**Affiliations:** 1 Department of Pathology, School of Medicine, National and Kapodistrian University of Athens, 75 Mikras Asias Street, Goudi, GR-115 27 Athens, Greece; 2 Department of Pathology, Attikon Hospital, 1 Rimini Street, GR-124 62 Chaidari, Athens, Greece; 3 Department of Pathology, Alexandra Hospital, 80 Vasilissis Sofias Street, GR-115 28 Athens, Greece

## Abstract

**Introduction:**

Our aim was to study the expression pattern of tissue inhibitor of metalloproteinases (TIMP)-3 protein in invasive breast carcinoma, and its clinicopathological and prognostic value as well as its relation to markers indicative of the tumor phenotype.

**Methods:**

Immunohistochemistry was performed on paraffin-embedded tissue specimens from 173 invasive breast carcinomas to detect the proteins TIMP-3, estrogen receptor (ER), progesterone receptor, p53, c-erbB-2, topoisomerase IIα and Bcl-2.

**Results:**

TIMP-3 protein was immunodetected in the cytoplasm of the malignant cells and the peritumoral stroma, as well as in *in situ *carcinoma and normal epithelium. Reduced expression of TIMP-3 protein within cancer cells was correlated with carcinomas of high nuclear and histological grade (*p *= 0.032 and *p *= 0.015, respectively), and low ER expression (*p *= 0.053). Moreover, TIMP-3 immunopositivity was inversely correlated with the expression of p53 and topoIIα proteins (*p *= 0.002 and *p *= 0.008, respectively), whereas it was positively associated with Bcl-2 expression (*p *= 0.020). Reduced expression of TIMP-3 protein within cancer cells was found to have an unfavorable impact on disease-free survival (*p *= 0.052) in the entirety of the patient population, as well as in both subgroups of lymph-node-positive and mutant-p53-negative patients (*p *= 0.007 and *p *= 0.037, respectively). Stromal localization of TIMP-3 protein was found to have no clinicopathological or prognostic value.

**Conclusion:**

This is the first immunohistochemical study to show that TIMP-3 protein within cancer cells is associated with tumor phenotype. Reduced expression of TIMP-3 protein within cancer cells was found to correlate with an aggressive tumor phenotype, negatively affecting the disease-free survival of both subgroups of lymph node-positive and mutant-p53-negative patients.

## Introduction

Matrix metalloproteinases (MMPs) consist of a family of proteases that have a major role in the remodeling and turnover of the extracellular matrix, which is involved in many physiological and pathological conditions including tumor growth and metastasis [1]. Tissue inhibitors of metalloproteinases (TIMPs) are natural MMP inhibitors and impede the proteolytic activity of MMPs by forming noncovalent 1:1 stoichiometric complexes [2]. Disruption of the balance between MMPs and TIMPs may influence invasion and metastasis of cancer and may thus modify patient outcome [1]. However, TIMPs exhibit several other biological functions in addition to the inhibition of active MMPs. These include the regulation of pro-MMP activation, tumor angiogenesis, cell growth and apoptosis [3].

TIMP-3 is one of the four known TIMPs (TIMP-1, TIMP-2, TIMP-3 and TIMP-4). It is known to be tightly bound to the matrix through interaction with heparan sulphate [4] The gene encoding TIMP-3 is a cell-cycle-regulated gene [5] whose promoter has been shown to be regulated by cell-cycle-related factors such as p53 [6]. *In vitro *studies have shown that TIMP-3 can suppress invasion [7-9] and may either promote [10] or inhibit [7,11] cell growth as well inducing apoptosis in cancerous [8,9,11,12] and non-cancerous [7] cells. Specifically, the latter is associated with death receptor modulation [12,13] and type II apoptotic pathway activation [12]. Interestingly, at least in the mouse, it has been demonstrated that the absence of TIMP-3 is important in regulating apoptosis in physiological processes [14]. Moreover, delivery of TIMP-3 to tumor xenografts has confirmed its tumor suppressive activity [11,15]. Studies on silencing of TIMP-3 by gene methylation also suggested a tumor suppression role in several malignancies [16].

TIMP-3 expression has been detected in several cancer types, including esophageal [17,18], colorectal [19], endometrial [20] and prostatic [21] cancer. However, in breast cancer, TIMP-3 mRNA was found by *in situ *hybridization to be expressed predominantly in the peritumoral stroma [22], whereas its clinicopathological and prognostic value has been evaluated in relation to the expression levels of TIMP-3 mRNA and measured by methods that did not distinguish the cancerous from the stromal origin of the transcripts [23,24].

The purpose of the present study was to investigate the expression pattern of TIMP-3 protein in invasive breast carcinoma to determine the clinicopathological and prognostic value of its various localizations and their relation to the tumor's phenotype through their association with biological indicators, such as the cell cycle-related proteins p53 and topoisomerase IIα (topoIIα), c-erbB-2 and the anti-apoptotic protein Bcl-2.

## Materials and methods

### Patients and samples studied

A total of 173 paraffin blocks with tumor samples were available from patients with resectable breast cancer, who had undergone surgery. We selected only women with histologically proven, clearly invasive breast carcinomas, regardless of their initial stage, in whom axillary lymph node dissection had been performed and who had all their resected materials studied histologically. The patients were aged from 25 to 86 years (mean 56.89 years). None of them had received radiation or chemotherapy preoperatively. The material acquired from them was used in research after informed consent had been obtained.

Routine histological examination was performed with hematoxylin–eosin staining. All carcinomas were classified in accordance with the criteria of the World Health Organization [25] and were recorded as invasive ductal or invasive lobular. The combined histological grade (1, 2 or 3) of infiltrating ductal carcinoma was obtained according to a modified Scarff–Bloom–Richardson histological grading system with guidelines as suggested by Nottingham City Hospital pathologists [26]. Nuclear grading was based on nuclear pleomorphism. Staging at the time of diagnosis was based on the TNM system [27]. Tumor size (less than 2 cm, 2 to 5 cm, more than 5 cm) and lymph node status were evaluated separately. The clinicopathological characteristics of the series are shown in Table 1. During the immunohistochemical procedure some specimens were destroyed, whereas others were considered to have too little tissue to be evaluated. The samples that were finally included in the statistical evaluation therefore numbered 167.

Follow-up was available for 166 patients, of whom 39 died of breast cancer and 109 had a recurrence. Mean survival time was 96.7 months (range 5 to 135 months) and median survival time was 111 months. Patient outcome was defined as disease-specific overall survival and recurrence-free survival, from surgery. All patients received conventional postoperative treatment depending on the extent of the disease, including radiation therapy and medical antiestrogen therapy, when indicated. Premenopausal patients with axillary involvement were treated with six courses of adjuvant chemotherapy.

### Immunohistochemistry

Immunohistochemical staining for TIMP-3 was performed on formalin-fixed paraffin sections 4 μm thick after heating overnight at 37°C and subsequent deparaffinization in xylene and rehydration through graded alcohols. After the quenching of endogenous peroxidase activity with 0.3% hydrogen peroxide in Tris-buffered saline for 30 minutes, we proceeded to microwave-mediated antigen retrieval in 10 mM citrate buffer (pH 6.0) at 750 W for 15 minutes (three cycles, 5 minutes each). After rinsing with Tris-buffered saline, normal horse serum was applied for 30 minutes to block non-specific antibody binding. Subsequently, sections were incubated overnight at 4°C with the primary antibodies. A two-step technique (Envision; Dako, Glostrup, Denmark) was used for visualization, with diaminobenzidine as a chromogen. Finally, sections were counterstained with hematoxylin and mounted.

A rabbit polyclonal antibody against the C terminus of human TIMP-3 (no. RB-1541; Neomarkers, Fremont, CA, USA) was used at a dilution of 1:70. For the detection of estrogen receptor (ER) and progesterone receptor (PR), p53, c-erbB-2, Bcl-2 and topoisomerase IIα proteins we used the following antibodies overnight, in a process similar to the aforementioned: anti-ER, clone 1D5 (dilution 1:450; Dako); anti-PR, clone IA6 (dilution 1:150; Dako); anti-p53, clone BP 53.12.1 (dilution 1:50; Oncogene, Cambridge, MA, USA); anti-c-erbB-2, clone CB11 (dilution 1:150; Biogenex, San Ramon, CA, USA); anti-Bcl-2, clone 124 (dilution 1:120; Dako, Glostrup, Denmark); and anti-topoIIα, clone JH2.7 (dilution 1:100; Biocare Medical, Walnut Creek, CA, USA).

### Evaluation of immunohistochemistry

Evaluation of the immunohistochemical staining was performed independently by two pathologists through light microscopic observation and without knowledge of the clinical data for each patient. Cases of disagreement were reviewed jointly to reach a consensus score. The score was the average from 10 distinct high-power fields observed under ×400 magnification. As positive controls we used both a placenta section previously known to be TIMP-3 immunoreactive (external control) as well as normal ductal epithelial cells and *in situ *components adjacent to cancer tissues (internal staining control). These in particular stained with high intensity, comparable to that of the external staining control. Negative controls had the primary antibody omitted and replaced by nonimmune normal serum from the same species as the primary antibody or by Tris-buffered saline.

TIMP-3 was detected in the cytoplasm of the malignant cells and the peritumoral stroma. Staining intensity and the number of stained cells were taken into consideration all through the evaluation process, and cytoplasmic staining in tumor cells and the peritumoral fibroblasts was scored on a scale of 0 to 3 in half steps, as described previously [28]. A score of 0 was given when no staining was detected, 1 if there was weak to moderate staining in less than 10% of cells, 2 if moderate to strong staining was present in 11 to 50% of cells, and 3 if strong staining in more than 50% of cells was detected. TIMP-3 was strongly expressed in internal positive controls. TIMP-3 protein staining in tumor cells fell into two distinct groups: those with expression equal to or reduced in relation to the internal positive staining controls. Tumor TIMP-3 staining was separated into these categories for the purposes of statistical analysis.

Staining for ER and PR was evaluated semiquantitatively with the H score system; a score of more than 50 was considered positive for both antigens [29]. Evaluation of the immunostaining of p53, c-erbB-2, Ki67 and topoIIa proteins was performed as described previously [30,31]. Because topoIIα immunopositive cells were rare, the percentage of 500 neoplastic cells that were positive for topoIIα was determined by image analysis [30].

### Statistics

The significance of the relationship between the expression of TIMP-3 and clinicopathological parameters was evaluated by univariate analysis with a χ^2 ^test and Fisher's exact probability test. TopoIIα expression failed to fit the Gaussian distribution. We therefore used nonparametric analysis of variance with ranks to assess the relationship of the aforementioned markers to topoIIa. The effect of TIMP-3 differential expression on postoperative survival rates was assessed with both univariate (log-rank test) and multivariate (stepwise forward Cox's proportional-hazards regression model) analysis; *p *≤ 0.05 was considered statistically significant.

## Results

**Figure 1:**
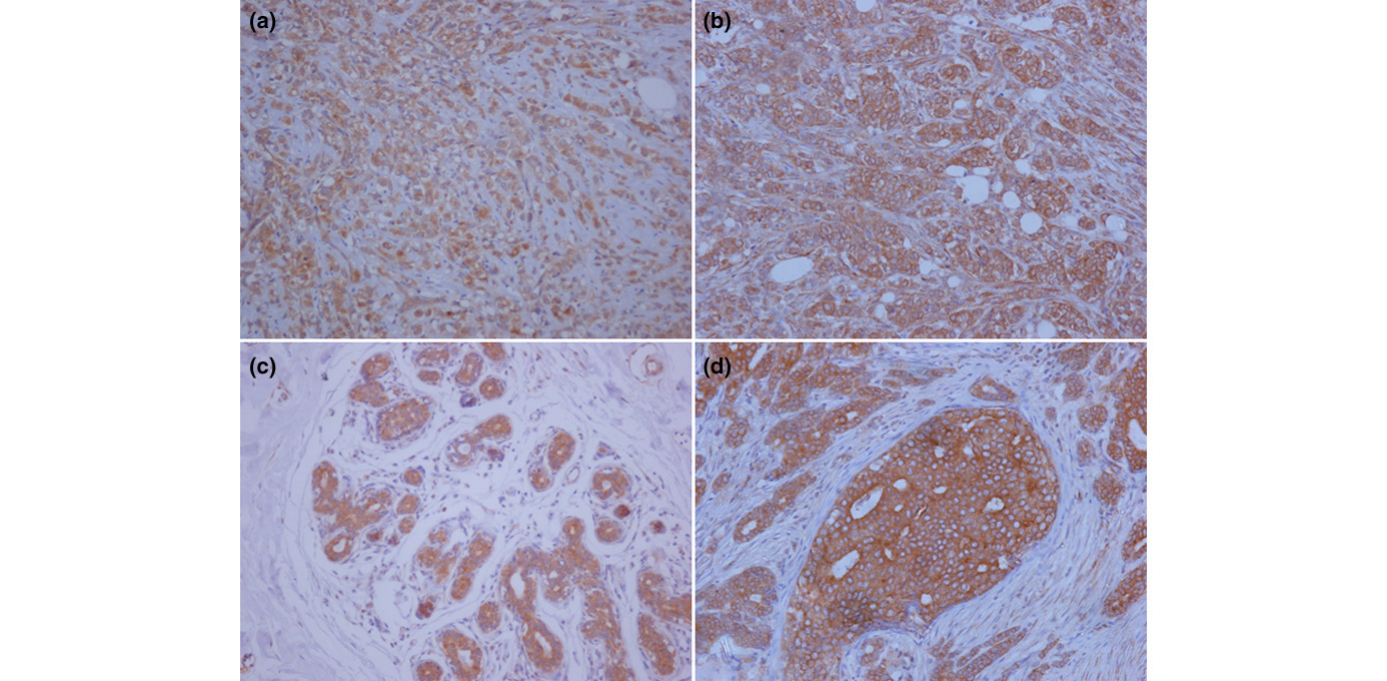
TIMP-3 protein expression in breast cancer and normal epithelium. The micrographs show tissue inhibitor of metalloproteinases (TIMP)-3 in the cytoplasm of a lobular carcinoma **(a)**, both the cancer cells and peritumoral stroma of a ductal carcinoma **(b)**, *in situ *carcinoma **(c) **and normal epithelium **(d)**. Envision/horseradish peroxidase staining; original magnification ×200.

**Figure 2:**
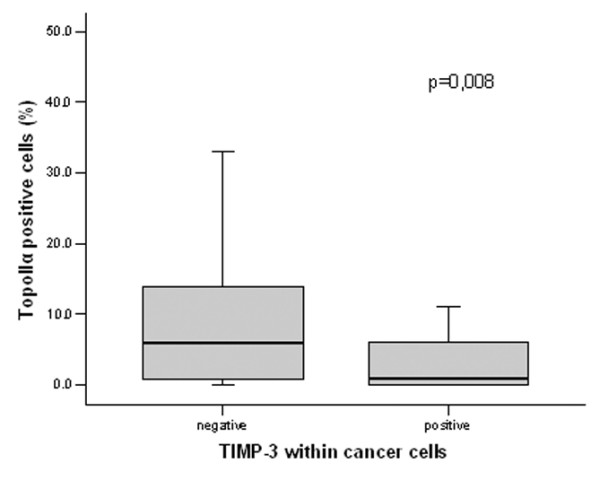
Relation between the expression of TIMP-3 and topoIIα proteins. Graphic representation of the relationship between tissue inhibitor of metalloproteinases (TIMP)-3 in cancer cells and topoIIα protein expressions.

TIMP-3 protein was immunodetected in the cytoplasm of the malignant cells and the peritumoral fibroblasts in 35.9% and 56.4% of cases, respectively (Figure 1a, b). In the cases where *in situ *carcinoma or normal epithelium were present, they demonstrated an intense immunopositivity for TIMP-3 (Figure 1c, d). Reduced expression of cancerous TIMP-3 protein was correlated with carcinomas of high nuclear and histological grades (*p *= 0.032 and *p *= 0.015, respectively). Moreover, TIMP-3 expression was weakly associated with ER expression (*p *= 0.053), whereas no correlation was found between cytoplasmic TIMP-3 and the rest of the clinicopathological parameters (Table 1). Tumor-cell-associated TIMP-3 was inversely correlated with p53 (*p *= 0.002) and topoIIα (*p *= 0.008, Figure 2), and was positively correlated with Bcl-2 expression (*p *= 0.020). No correlation between TIMP-3 and c-erbB-2 status was observed (Table 1).

**Figure 3:**
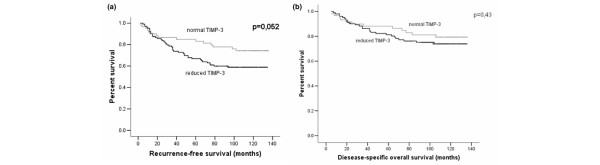
The prognostic significance of TIMP-3 protein expression in the entire patient population. The graphs show the effect of tissue inhibitor of metalloproteinases (TIMP)-3 expression on patient recurrence-free **(a) **and disease-specific overall **(b) **survival (log-rank test).

**Figure 4:**
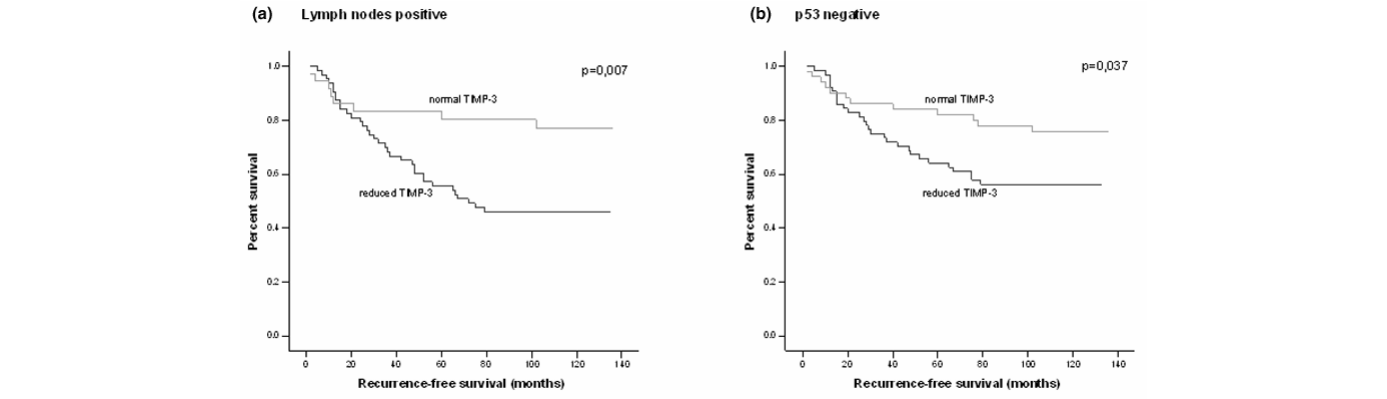
The prognostic significance of TIMP-3 protein expression in the LN^+ ^and p53^- ^patient subgroups. The graphs show the impact of tissue inhibitor of metalloproteinases (TIMP)-3 on the recurrence-free survival of the lymph-node (LN)-positive **(a) **and mutant-p53-negative **(b) **patients (log-rank test).

With regard to patient outcome, reduced TIMP-3 expression within cancer cells was weakly correlated with reduced recurrence-free survival in the overall patient population by univariate analysis (*p *= 0.052; Figure 3a). Moreover, reduced TIMP-3 in cancer cells was associated with reduced recurrence-free survival in the 100 lymph-node-positive cases (*p *= 0.007; Figure 4a) and in the subset of 118 p53-negative cancers (*p *= 0.037; Figure 4b). However, cancer-cell-associated TIMP-3 was not prognostic for disease-specific overall survival in the entire patient population (Figure 3b) or in any patient subset (data not shown). Nor was TIMP-3 prognostic after multivariate analysis. Indeed, only tumor stage was an independent indicator for recurrence-free (hazard ratio 4.0, 95% confidence interval 2.3 to 7.1, *p *< 0.0001) or disease-specific overall survival (hazard ratio 5.0, 95% confidence interval 2.5 to 10.1, *p *< 0.0001) after adjusting for age, therapy received and all other factors listed in Table 1. Unlike cancer-cell-associated TIMP-3, TIMP-3 expression within fibroblasts had no prognostic power (data not shown), nor was it associated with any clinicopathological factor (Table 1).

## Discussion

This is the first immunohistochemical study investigating the clinicopathological and prognostic significance of the various localizations of TIMP-3 protein expression. In the present study, TIMP-3 protein was immunodetected in the cytoplasm of both malignant cells and peritumoral fibroblasts. This is consistent with studies that reported the same localizations in esophageal [17], colorectal [19] and endometrial cancers [20]. Moreover, the fact that we detected TIMP-3 protein in a larger percentage of cases in the peritumoral fibroblasts than in malignant cells agrees with Byrne and colleagues [22] who, by *in situ *mRNA hybridization, found TIMP-3 gene to be predominantly expressed by fibroblastic cells within the tumor stroma. Furthermore, we found TIMP-3 protein to be expressed in both normal epithelium and the *in situ *component, where they existed adjacent to the cancerous tissue. This is consistent with the observation of Darnton and colleagues [17], who immunodetected TIMP-3 in normal and metaplastic esophageal epithelium as well as in esophageal adenocarcinoma.

In the present study, reduced expression of TIMP-3 protein was correlated with tumors of higher nuclear and histological grades; in other words, tumors of poor differentiation that usually display a more aggressive biological behavior. This is the first study to report a relationship between TIMP-3 expression and the grade of tumor differentiation. To our knowledge, only two other studies have investigated TIMP-3 expression in breast cancer [23,24] and they found no correlation with any clinicopathological parameter. This discrepancy from our findings may be assigned to the different methodology used. Specifically, both groups of authors used polymerase chain reaction to assess TIMP-3 mRNA expression levels in tumor tissue, without distinguishing whether mRNA comes from the malignant cells or the surrounding stromal cells.

The aforementioned association of the reduced expression of TIMP-3 protein with an aggressive tumor phenotype is further supported by its correlation with the expression of the mutant p53 and topoIIα proteins. The wild-type gene encoding p53 is a tumor suppressor gene whose protein product regulates the cell cycle checkpoint in response to DNA damage [32]. Mutations in this gene, which deactivate its growth-suppressing activities, have been observed in most human tumors. Here it is considered to participate not only in the acquisition of the transformed phenotype but also in the process of cancer progression from benign to metastatic [33]. The inverse correlation between TIMP-3 protein expression and mutant p53 is consistent with the studies that have shown that expression of the gene encoding TIMP-3 is repressed in cells expressing mutant p53 alleles [6].

TopoIIα is a proliferation marker that is considered to estimate the number of actively cycling cells [34]. The inverse association of TIMP-3 protein with a proliferation marker is in line with its inverse correlation with nuclear grade, because the number of mitoses is coevaluated in the assessment of tumor's nuclear grade. Moreover, it agrees with studies that have shown TIMP-3 overexpression to inhibit the increase in cell number *in vitro *[7,11]. In other words, according to our findings, reduced TIMP-3 expression seems to be related to cell cycle deregulation and tumor cell proliferation, further contributing to the development of an aggressive tumor phenotype.

Bcl-2 is a negative regulator of apoptosis-specific mitochondrial functions [35]. In the present study we found a positive correlation between TIMP-3 protein expression and the antiapoptotic index Bcl-2, thus suggesting the involvement of TIMP-3 protein in apoptosis suppression. Contrary to our findings, TIMP-3 has been shown to promote apoptosis [8,9,11-13] in an MMP-independent mechanism [11]. However, and in accordance with our findings, TIMP-3^-/- ^mice have been shown to display accelerated postlactational epithelial cell apoptosis [14], suggesting that the presence of TIMP-3 in the mammary gland is presumably associated with reduced apoptosis.

Moreover, in the present study we discovered a positive correlation between TIMP-3 protein within cancer cells and ER. This finding is in line with that of Span and colleagues [23] and Kotzsch and colleagues [24], who also reported TIMP-3 mRNA levels to be higher in steroid hormone receptor-positive samples, and yet not in accord with other studies that demonstrated that estradiol induced a decrease in TIMP-3 in uterine tissue [36] or nulliparous murine mammary gland [37].

As regards survival rate, decreased expression of TIMP-3 within the cytoplasm of cancer cells was associated with poor disease-free survival of our patients. This is in accord with the observation that overexpression of TIMP-3 in tumor cells inhibits invasion, a function consistent with its role as an MMP inhibitor, and induces apoptosis *in vitro *[8,9]. Our finding was of weak statistical significance and failed to be confirmed in multivariate analysis, differing from both Span and colleagues [23], who found no prognostic significance for TIMP-3 mRNA levels, and Kotzsch and colleagues [24], who showed low TIMP-3 mRNA levels to be an independent poor prognosticator of patient disease-free survival. This may be due to the differences in the methods used or it may reflect the weak rather than the strong prognostic power of TIMP-3 and the modest number of cases examined. However, in subgroup analyses, decreased immunoexpression of TIMP-3 protein was found to be a significant prognostic indicator of an unfavorable recurrence-free survival in both subgroups of lymph-node-positive and mutant-p53-negative patients. This probably suggests that TIMP-3, as an invasion suppressor, may have a major role in patients with positive lymph nodes in whom the invasive process has been already activated. Moreover, the fact that in our study TIMP-3 was weakly associated with outcome but was fairly strongly associated with p53 status, in combination with the studies suggesting that p53 suppresses TIMP-3 expression, makes one think that TIMP-3 association with outcome may merely be a reflection of p53 status rather than any causal effects that it might have on cancer progression. In other words, p53 may be driving both progression and tumor-associated TIMP-3 expression. However, it is notable that TIMP-3 was still prognostic in the p53-negative subset of cases, suggesting that its correlation with outcome may not merely reflect p53 status but may indeed have an active role in suppressing cancer progression.

Finally, the fact that TIMP-3 protein within fibroblasts was found to have no clinicopathological or prognostic value indicates that, at least in our series of tumor samples, TIMP-3 within cancer cells seems to be more strongly correlated with tumor biological behavior than stromal TIMP-3. Besides, although TIMP-3 has been shown to be bound to the extracellular matrix, to our knowledge there are no studies to elucidate the locations at which TIMP-3 protein takes action.

## Conclusion

This is the first immunohistochemical study to show that, of the two tissue localizations of TIMP-3 protein, cancer and stromal, the one within the cancer cells seems to have a more active role in the modulation of tumor phenotype. Thus, decreased expression of TIMP-3 protein within cancer cells seems to be associated with an aggressive tumor phenotype through its correlations with nuclear and histological grade, expression of the proliferation marker topoIIα and expression of the apoptosis-related indicators, mutant p53 and Bcl-2. Moreover, reduced expression of TIMP-3 was found to be a significant prognostic indicator of shortened disease-free survival in the lymph-node-positive and mutant-p53-negative patients, indicating that immunodetection of TIMP-3 protein could improve the prediction of disease recurrence in some subgroups of patients with invasive breast cancer.

## Abbreviations

ER = estrogen receptor; MMP = matrix metalloproteinase; PR = progesterone receptor; TIMP = tissue inhibitor of metalloproteinases.

## Competing interests

The authors declare that they have no competing interests.

## Authors' contributions

EM participated in the design of the study, carried out the immunohistochemical method, performed the statistical analysis and drafted the manuscript. CM and GA classified the invasive carcinomas and carried out the evaluation of the immunohistochemical staining. IG and LN participated in the design and the coordination of the study. SM and AK collected the human tissue. All authors read and approved the final manuscript.

## Figures and Tables

**Table 1:** Correlation of TIMP-3 protein expression with clinicopathological parameters and various biological markers.

Parameter	Total	Preserved expression					
		
		TIMP-3 within cancer cells			Fibroblastic TIMP-3		
		*n*	%	*p*	*n*	%	*p*
Menopausal status							
Before	52	19	36.5	NS	25	48.1	NS
After	115	41	35.7		53	46.1	
Histological type							
Ductal	133	46	34.6	NS	61	45.9	NS
Lobular	33	14	42.4		17	51.5	
Histological grade							
1	21	12	57.1	0.015	12	54.5	NS
2	98	36	36.7		44	44.9	
3	43	9	20.9		19	44.2	
Nuclear grade							
1	58	28	48.3	0.032	27	46.6	NS
2	55	19	34.5		25	44.6	
3	53	13	24.5		26	49.1	
Tumor size							
<2	39	19	48.7	NS	20	51.3	NS
2 to 5	101	35	34.7		43	42.2	
>5	26	6	23.1		15	57.7	
LN status							
Not infiltrated	65	24	36.9	NS	30	45.5	NS
Infiltrated	100	36	36.0		48	48.0	
Stage							
1	29	13	44.8	NS	15	51.7	NS
2	108	40	37.0		47	43.1	
3	28	7	25.0		16	57.1	
ER status							
Negative	75	21	28.0	0.053	35	46.7	NS
Positive	91	39	42.9		43	46.7	
PR status							
Negative	84	28	33.3	NS	40	47.6	NS
Positive	82	32	39.0		38	45.8	
p53 status							
Negative	118	51	43.2	0.002	57	47.9	NS
Positive	46	8	17.4		19	41.3	
Bcl-2 status							
Negative	54	16	29.6	0.02	29	53.7	NS
Positive	79	40	50.6		41	51.3	
c-erbB-2 status							
Negative	62	18	29.0	NS	25	39.7	NS
Positive	104	42	40.4		53	51.0	

ER, estrogen receptor; LN, lymph node; NS, not significant; PR, progesterone receptor; TIMP, tissue inhibitor of metalloproteinases.
